# Leveraging glycomics data in glycoprotein 3D structure validation with Privateer

**DOI:** 10.3762/bjoc.16.204

**Published:** 2020-10-09

**Authors:** Haroldas Bagdonas, Daniel Ungar, Jon Agirre

**Affiliations:** 1 York Structural Biology Laboratory, Department of Chemistry, University of York, Wentworth Way, York, YO10 5DD, UK; 2 Department of Biology, University of York, Wentworth Way, York, YO10 5DD, UK

**Keywords:** electron cryomicroscopy, glycoinformatics, glycomics, Privateer, X-ray crystallography

## Abstract

The heterogeneity, mobility and complexity of glycans in glycoproteins have been, and currently remain, significant challenges in structural biology. These aspects present unique problems to the two most prolific techniques: X-ray crystallography and cryo-electron microscopy. At the same time, advances in mass spectrometry have made it possible to get deeper insights on precisely the information that is most difficult to recover by structure solution methods: the full-length glycan composition, including linkage details for the glycosidic bonds. The developments have given rise to glycomics. Thankfully, several large scale glycomics initiatives have stored results in publicly available databases, some of which can be accessed through API interfaces. In the present work, we will describe how the Privateer carbohydrate structure validation software has been extended to harness results from glycomics projects, and its use to greatly improve the validation of 3D glycoprotein structures.

## Introduction

**Figure 1:**
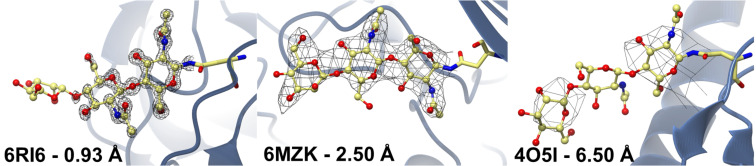
Comparison of the glycan features in electron density maps over a range of resolutions from selected glycoprotein structures (PDB entries: 6RI6 [19]; 6MZK [20]; 4O5I [21]). The electron density maps were obtained with X-ray crystallography. The data resolution and PDB entry IDs associated with the structures have been directly annotated on the structure. Left: A high-resolution example where monosaccharides and the conformations can be elucidated; middle: A medium resolution example where the identification starts to become difficult; right: A low-resolution example for which all prior knowledge must be used. Despite coming from different glycoprotein structures, the glycan has the same composition, and thus is assigned a unique GlyTouCan ID of G15407YE.

Glycosylation-related processes are prevalent in life. The attachment of carbohydrates to macromolecules extends the capabilities of cells to convey significantly more information than what is available through protein synthesis and the expression of the genetic code alone. For example, glycosylation is used as a switch to modulate protein activity [1]; glycosylation plays a crucial part in folding/unfolding pathways of some proteins in cells [2–3]; the level of *N*-glycan expression regulates the adhesiveness of a cell [4]; glycosylation also plays a role in immune function [5] and cellular signalling [5–6]. At the forefront, glycosylation plays a significant role in influencing protein–protein interactions. For example, the influenza virus uses the haemagglutinin glycoprotein to recognise and bind sialic acid decorations of human cells in the respiratory tract [7]. Glycosylation is also used by pathogens to evade the host’s immune system via glycan shields [8–10], and thereby to delay an immune response [11]. The structural study of these glycan-mediated interactions can provide unique insight into the molecular interplay governing these processes. In addition, it can provide structural snapshots in atomistic detail that can be used to generate molecular dynamics simulations describing a wider picture underpinning glycan and protein interactions [12]. Unfortunately, significant challenges have affected the determination of glycoprotein structures for decades and have had a detrimental impact on the quality and reliability of the produced models. Anomalies have been reported regarding carbohydrate nomenclature [13], glycosidic linkage stereochemistry [14] and torsion [15–16], and most recently, ring conformation [17]. Most of these issues have now been addressed as part of ongoing efforts to provide better software tools for structure determinations of glycoproteins, although the most difficult cases remain hard to solve. Chiefly among these is the scenario where the experimentally resolved electron density map provides evidence of glycosylation, without enough resolution to derive definite and comprehensive details about the structural composition of the oligosaccharides (Figure 1). Glycan microheterogeneity and the lack of carbohydrate-specific modelling tools have often been named as the principal causes for these issues [18].

### Heterogeneity of glycoproteins

Unlike protein synthesis, which is encoded in the genome and follows a clear template, glycan biosynthesis is not template-directed. A single glycoprotein will exist in multiple possibilities of products that can emerge from the glycan biosynthesis pathways, and these are known as glycoforms [22]. More specifically, the variation can appear in terms of which potential glycosylation sites are occupied at any time – macroheterogeneity – or variations in the compositions of the glycans added to specific glycosylation sites – microheterogeneity. This variation in the microheterogeneous composition patterns arises due to the competition of glycan-processing enzymes in biosynthesis pathways [23].

### Implications for the structure determination of glycoproteins

Several experimental techniques can be used to obtain 3D structures of glycoproteins: X-ray crystallography (MX, which stands for macromolecular crystallography), nuclear magnetic resonance spectroscopy (NMR) and electron cryomicroscopy (cryo-EM). As of publication date, the overwhelming majority of glycoprotein structures have been solved using MX [24–25].

The biggest bottleneck in MX is the formation of crystals of the target macromolecule or complex. The quality of the crystal directly determines the resolution – a measure of the detail in the electron density map. Homogenous samples at high concentrations are required to produce well-diffracting crystals [26]. Samples containing glycoprotein molecules do not usually fulfill this criterion. More often than not, MX falls short at elucidating carbohydrate features in glycoproteins due to glycosylated proteins being inherently mobile and heterogeneous [22]. Moreover, oligosaccharides often significantly interfere with the formation of crystal contacts that allow the formation of well-diffracting crystals. Because of this, glycans are often truncated in MX samples to aid crystal formation [27].

In cryo-EM, samples of glycoproteins are vitrified at extremely low temperatures rather than crystallised, as in MX. The rapid cooling of the sample allows to capture snapshots of the molecules at their various conformational states, and thus potentially maintaining glycoprotein states more closely to their native environments in comparison to crystallography [28]. Nevertheless, cryo-EM is still not an end-all solution to solving glycoprotein structures: the flexible and heterogeneous nature of glycans still has an adverse effect on the quality of the data, affecting the image reconstruction [29]. Moreover, due to the low signal-to-noise ratio, the technique works more easily with samples of a high molecular weight; this situation, however, is evolving rapidly, with reports of sub-100 kDa structures becoming more frequent lately [30–31]. Crucially, MX and cryo-EM can complement each other to counteract issues that both face individually [32].

The two techniques produce different information – electron density (MX) or electron potential (cryo-EM) maps – but the practical considerations in terms of the atomistic interpretation hold true for both: provided that at least the secondary structural features can be resolved in a 3D map, a more or less complete atomic model will be expected as the final result of the study. Modelling of carbohydrates into 3D maps can be more complex than modelling proteins [33], although recent advances in software are closing the gap [34–36]. However, to date it remains true that most model building software is protein-centric [15]. As a consequence, the glycan chains in glycoprotein models that have been elucidated before recent developments in carbohydrate validation and modelling software tend to contain a significant amount of errors: wrong carbohydrate nomenclature [13], biologically implausible glycosidic linkage stereochemistry [14], incorrect torsion [15–16], and unlikely high-energy ring conformations [17]. Early efforts in the validation of carbohydrate structures saw the introduction of online tools such as PDB-CARE [37] and CARP [16]; more recently, we released the Privateer software [24], which was the first carbohydrate validation tool available as part of the CCP4i2 crystallographic structure solution pipeline [38]. In its first release, Privateer was able to perform stereochemical and conformational validation of pyranosides, analyse the glycan fit to electron density map and offered tools for restraining a monosaccharide minimal-energy conformation.

While these features were recognised to address some long-standing needs in carbohydrate structure determination [39–40], significant challenges remain, particularly in the scenario where the glycan composition cannot be ascertained solely from the three-dimensional map. Unfortunately, this problematic situation happens frequently, especially in view of the fact that the median resolution for glycoproteins (2.4 Å) is lower than that of non-glycosylated – potentially including fully deglycosylated – proteins (2.0 Å) [41]. To date, only one publicly available model building tool has attacked this issue: the Coot software offers a module that will build some of the most common *N*-linked glycans in a semiautomated fashion [34]. Indeed, the Coot module was built around the suggestion that only the most probable glycoforms should be modelled unless prior knowledge of an alternative glycan composition exists in the form of, e.g., mass spectrometry data [14].

### Harnessing glycomics and glycoproteomics results to inform glycan model building

Current methods used to obtain accurate atomistic descriptions of molecules fall short in dealing with the heterogeneity of glycoproteins. However, there are other methods that have been proven to successfully tackle the challenges posed by glycan heterogeneity, with mass spectrometry emerging as the one with the most relevance due to the ability to elucidate the complete composition descriptions of individual oligosaccharide chains on glycoproteins [42].

The mass spectrometric analysis of glycosylated proteins can be with (glycomics) or without (glycoproteomics) the release of oligosaccharides from the glycoprotein. Usually, glycomics and glycoproteomics experiments are carried out together to obtain a complete description of the glycoprotein profile. Glycomics experiments are required to distinguish stereoisomers and the linkage information in order to obtain a full structural description about a glycan, whereas glycoproteomics are required to establish the glycan variability and occupancy at the glycosylation sites of the protein [43]. Typically, these analyses are based on mass spectrometry techniques, such as electrospray ionization mass spectrometry (ESIMS) and matrix-assisted laser desorption ionization MS (MALDIMS) [43]. Mass spectrometry techniques are best suited for the determination of the composition of monosaccharide classes and the chain length. However, the in-depth analysis of a glycan typically requires the integration of complementary analytic techniques, such as nuclear magnetic resonance (NMR) and capillary electrophoresis (CE). Nevertheless, depending on the sample, advanced mass spectrometry techniques can be used to counteract the need for complementary analytic techniques. One of the examples of this is tandem mass spectrometry, where the glycan fragmentation is controlled to obtain the identification of the glycosylation sites and a complete description of the glycan structure compositions, including linkage and sequence information [44]. Moreover, recent advances in ion mobility mass spectrometry can now also be used for a complete glycan analysis [45].

The analysis and interpretation of mass spectrometry spectra produced by glycans is a challenge. Most significantly, in MS outputs, glycans appear in their generalized composition classes, i.e., Hex, HexNAc, dHex, NeuAc, etc. The identity elucidation of generalized unit classes into specific monosaccharide units (such as Glc, Gal, Man, GalNAc, etc.) requires prior knowledge of the glycan biosynthetic pathways [46]. Additional sources of prior knowledge are bioinformatics databases that have been curated through the deposition of experimental data. Bioinformatics databases contain detailed descriptions of the glycan compositions and *m*/*z* values of specific glycans, and therefore aiding the process of glycan annotation [47]. Such bioinformatics databases can usually be interrogated using textual or graphical notations that describe the glycan sequence. However, due to the glycan complexity and the incremental nature of the different glycomics projects, numerous notations have been developed over the years – e.g., CarbBank [48] utilized CCSD [48] and EuroCarbDB [49] and GlycomeDB [50] used GlycoCT [51] (Table 1).

**Table 1:** A comparison of the structural information storage capabilities of different sequence formats used in glycobioinformatics.^a^

notation	multiple connections	repeating units	alternative residues	linear notation	atomic ambiguity

CCSD(CarbBank)	–	+	–	+	–
LINUCS	–	+	–	+	–
GlycoSuite	–	–	+	+	–
BCSDB	(+)	(+)	+	+	–
LinearCode	–	–	+	+	–
KCF	+	+	–	–	–
GlycoCT	+	+	+	–	–
Glyde-II	+	+	–	–	–
WURCS 2.0	+	+	+	+	+

^a^“+” Denotes that information can be stored directly without any significant issues, “(+)” denotes that information can be stored indirectly, or that there are some issues and “–” denotes that information description in the particular sequence format is unavailable. This table is a simplified version of the one originally published by Matsubara et al. [52].

Thankfully, data from discontinued glycomics projects are not lost but were integrated into newer platforms, often with novel notations. One such example is GlyTouCan [53], which uses both GlycoCT [54] and WURCS [53] as notation languages. As a result, tools that interconvert between notations were developed to successfully integrate old data into new platforms. Additionally, the introduction of tools such as GlycanFormatConverter [55] to convert WURCS notations into more human-readable formats has eased the interpretation of glycan databases.

Significantly, the GlyTouCan project aims to create a public repository of known glycan sequences by assigning them unique identification tags. Each identification tag describes a glycan sequence in the WURCS notation, and this allows to link specific glycans to other databases, such as GlyConnect [56], UniCarb-DB [57] and others, any of which are tailored to specific flavours of glycomics and glycoproteomics experiments. Ideally, this implementation ends up requiring the user to be familiar with a single notation – WURCS – used to represent sequences of glycans.

### From glycomics/glycoproteomics to carbohydrate 3D model building and validation in Privateer

Many fields, for example pharmaceutical design and engineering [58], molecular dynamics simulations [59] and protein interaction studies [60], rely upon structural biology to produce accurate atomistic descriptions of glycoproteins. However, due to clear limitations of elucidating carbohydrate features in MX/cryo-EM electron-density maps, structural biologists are likely to make mistakes. This introduces the possibility of modelling wrong glycan compositions in glycoprotein models, going as far as not conforming with general glycan biosynthesis knowledge. Model building pipelines would therefore greatly benefit from the ability to validate against the knowledge of glycan compositions elucidated via glycomics/glycoproteomics experiments. This warrants the need for new tools that are able to link these methodologies, through an intermediate interconversion library.

A foundation for such interconversion libraries exists in the form of the carbohydrate validation software Privateer. The program is able to compute individual monosaccharide conformations from a glycoprotein model, check whether the modelled carbohydrates atomistic definitions match dictionary standards as well as output multiple helper tools to aid the processes of refinement and model building [24]. Most importantly, Privateer already contains methods that allow the extraction of carbohydrate atomistic definitions to create abstract definitions of glycans in memory, and thus already laying a foundation for the generation of unique WURCS notations and providing a straightforward access to bioinformatics databases that are integrated in the GlyTouCan project.

## Methods

The algorithm used to generate the WURCS notation in Privateer is based on the description published in Tanaka et al. [61], with required updates applied from Matsubara et al. [52]. WURCS was designed to deal with the incomplete descriptions of glycan sequences emerging from glycomics/glycoproteomics experiments (i.e., undefined linkages, undefined residues and ambiguous structures in general). However, the lack of this detail is unlikely to be supported in “pdb” or “mmCIF'” format files, which are a standard in structural biology. As a result, the “atomic ambiguity” capability (Table 1) is not supported in Privateer’s implementation. Moreover, Privateer’s implementation of WURCS relies on a manually compiled dictionary that translates the PDB Chemical Component Dictionary [62] three-letter codes of carbohydrate monomer definitions found in the structure files into WURCS definitions of unique monomers (described as “UniqueRES” [52]).

The WURCS notations are generated for all detected glycans that are linked to protein backbones in the input glycoprotein model. For every glycan chain in the model, the algorithm computes a list of all detected monosaccharides that are unique and stores that information internally in memory. Then, the algorithm calculates the unit counts in a glycan chain – how many unique monosaccharides are modelled in the glycan chain, the total length of the glycan chain and computes the total number linkages between monosaccharides. After the composition calculations are carried out, the algorithm begins the generation of the notation by printing out the unit counts. Then, the list of unique monosaccharide definitions in the glycan chain are printed out by converting the three-letter PDB codes into WURCS-compliant definitions. Afterwards, each individual monosaccharide of the glycan is assigned a numerical ID according to its occurrence in the list of unique monosaccharides. Finally, the linkage information between monosaccharide pairs are generated by assigning individual monosaccharides a unique letter ID according to their position in the glycan chain. Alongside a unique letter ID, a numerical term is added that describes a carbon position from which the bond is formed to another carbohydrate unit. Crucially, the linkage detection in Privateer does not rely at all on metadata present in the structure file. Instead, linkages are identified based on the perceived chemistry of the input model: which atoms are close enough – but not too close – to be plausibly linked.

**Figure 2:**
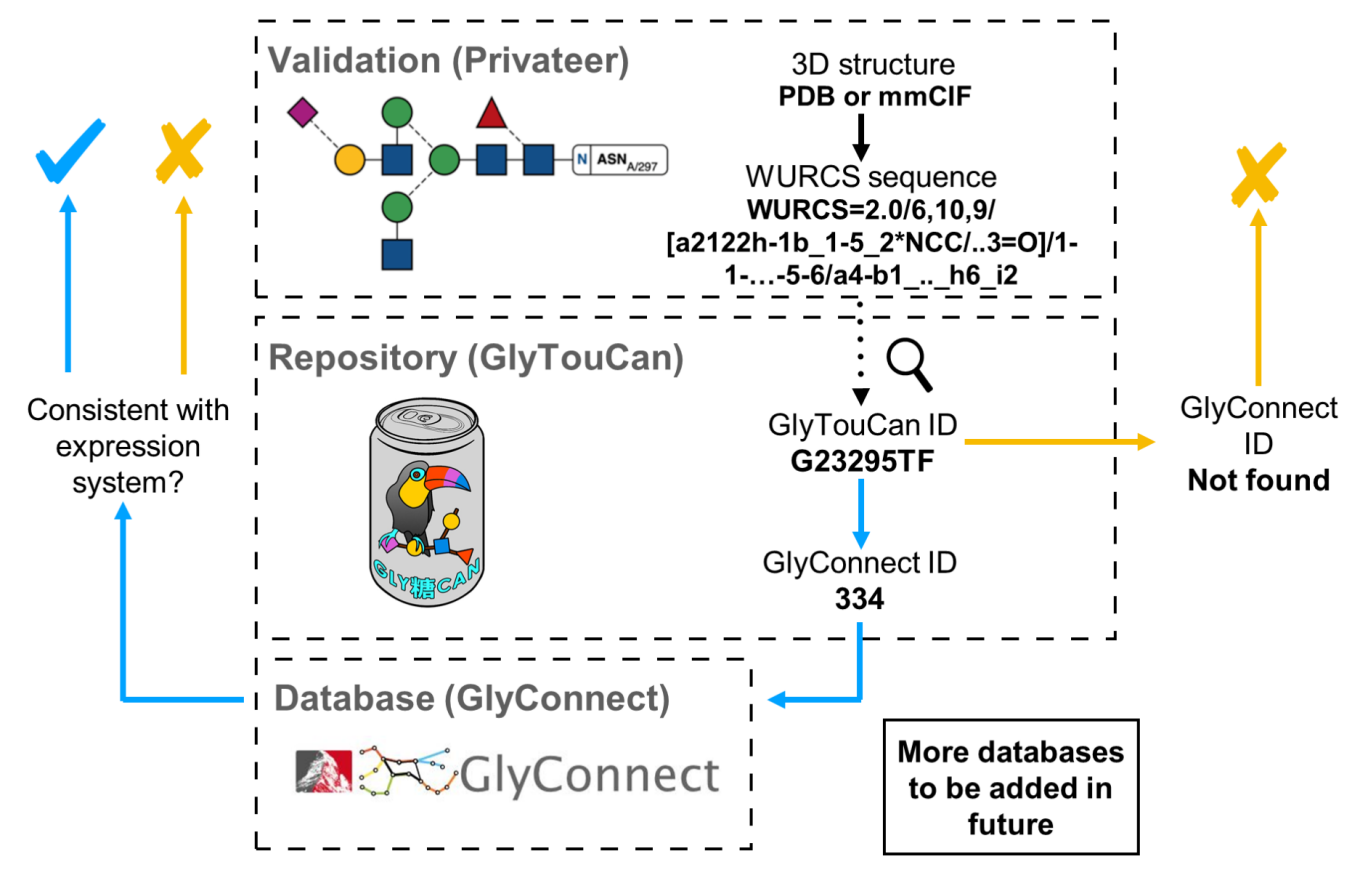
A roadmap of the software development project that allows structural biologists to quickly obtain detailed information about specific glycans in glycoprotein models from glycomics/glycoproteomics databases. The GlyTouCan (https://glytoucan.org/) and GlyConnect (https://glyconnect.expasy.org/) logos have been reproduced here under explicit permission from their respective authors.

The generated WURCS string can then be used to search whether an individual glycan chain has been deposited in GlyTouCan. The scan of the repository occurs internally within the Privateer software, as all the data is stored in a single structured data file written in JSON format that is distributed together with Privateer. If the existence of a glycan in the database is confirmed, then the software can attempt to find records about the sequence on other, more specialised databases (currently only GlyConnect) to obtain information such as the source organism, the type of glycosylation and the glycan core to carry out further checks in the glycoprotein model (Figure 2).

### Availability and performance of the algorithm

This new version of Privateer (MKIV) will be released as an update to CCP4 7.1. To demonstrate the capabilities of the computational bridge integrated in the newest version of Privateer (for standalone bundles, please refer to privateer branch “privateerMKIV_noccp4” of GitHub repository with the installation instructions provided in the README.md file [63]), it was run on all *N*-glycosylated structures in the PDB solved using MX and cryo-EM. The list of structures used in this demonstration was obtained from Atanasova et al. [18]. The computational analysis of the demonstration revealed a relatively small proportion of deposited glycoprotein models containing glycan chains that do not have a unique GlyTouCan accession ID assigned, raising questions about the provenance of their structures. Importantly, the majority of the glycan chains that do have a unique GlyTouCan accession ID assigned (except for single residues linked to protein backbones), have also been successfully matched on the GlyConnect database (Table 2).

**Table 2:** Comparison of the successful glycan matches detected by Privateer in the GlyTouCan and the GlyConnect database.^a^

experimental technique	glycan chain length	GlyTouCan ID found	GlyTouCan ID not found	% of GlyTouCan in GlyConnect	total glycan chains

MX	1	16797	0	1%	16797
MX	2	5870	5	90%	5875
MX	3	2550	17	71%	2567
MX	4	1012	21	80%	1033
MX	5	834	72	74%	906
MX	6	460	85	69%	545
MX	7	345	55	77%	400
MX	8	235	25	85%	260
MX	9	164	16	81%	180
MX	10	118	5	92%	123
MX	11	20	5	85%	25
MX	12	8	4	75%	12
MX	13	0	1	0%	1
MX	14	0	0	0%	0
MX	15	2	0	0%	2
MX	16	0	1	0%	1
cryo-EM	1	2080	0	3%	2080
cryo-EM	2	1081	0	98%	1081
cryo-EM	3	439	0	96%	439
cryo-EM	4	143	0	93%	143
cryo-EM	5	146	2	85%	148
cryo-EM	6	70	1	97%	71
cryo-EM	7	45	0	100%	45
cryo-EM	8	26	0	88%	26
cryo-EM	9	15	1	100%	16
cryo-EM	10	16	0	100%	16
cryo-EM	11	4	0	100%	4
cryo-EM	12	1	0	100%	1
cryo-EM	13	1	0	0%	1

^a^Glycans obtained from the glycoprotein models were elucidated by X-ray crystallography and cryo-EM.

## Results

### Examples of use

As observed in previous studies, glycoprotein models deposited in the PDB feature flaws ranging from minor irregularities to gross modelling errors [14,17,41,64]. The automated validation of minor irregularities was already possible with automated tools such as pdb-care [37], CARP [65], and Privateer [24]. However, the automated detection of gross modelling errors is currently a challenge due to the lack of publicly available tools. Our newly developed computational bridge between structural biology and glycomics databases makes the detection of gross modelling errors easier, as demonstrated by the following examples.

#### Example 1 – 2H6O

The glycoprotein model (PDB code 2H6O) proposed by Szakonyi et al. [66] contains 12 glycans, as detected by Privateer. The model became infamous after it sparked the submission of a critical correspondence published by Crispin et al. [14]. The article contained a discussion about the proposed model containing glycans that were previously unreported and inconsistent with glycan biosynthetic pathways. In particular, the model contained oligosaccharide chains with Man-(1→3)-GlcNAc and GlcNAc-(1→3)-GlcNAc linkages, β-galactosyl motifs capping oligomannose-type glycans and hybrid-type glycans containing terminal Man-(1→3)-GlcNAc [14]. Moreover, the proposed model contained systematic errors in the anomer annotations and carbohydrate stereochemistry. To this day, there is still no experimental evidence reported for these types of linkages and capping in an identical context.

**Figure 3:**
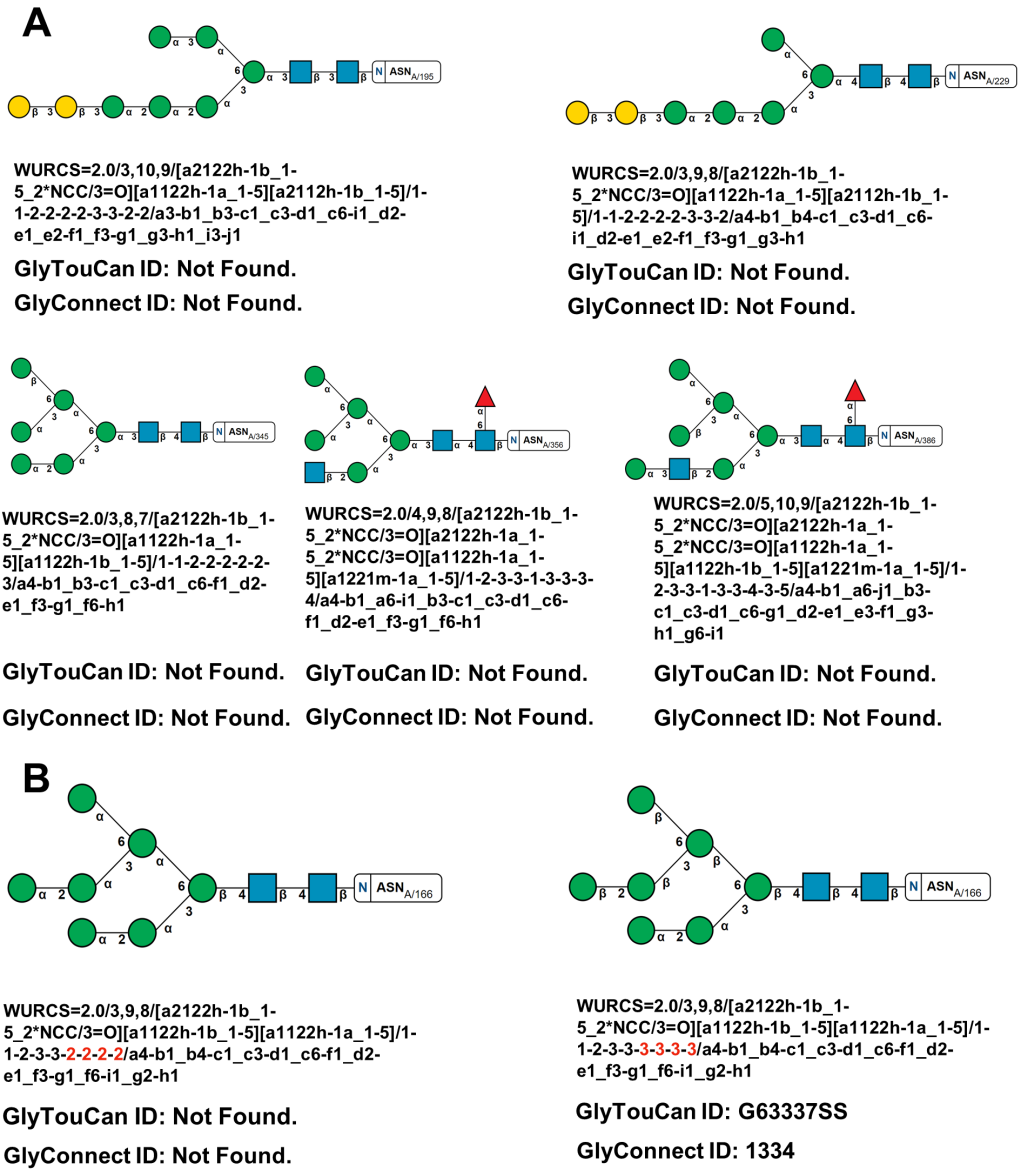
N-Linked glycans in Epstein Barr virus major envelope glycoprotein (PDB entry: 2H6O [66]). A) A selection of the glycan chains that failed to return database IDs with their WURCS sequences extracted from the Privateer CCP4i2 report. B) Glycan chain (right) for which a GlyTouCan and GlyConnect ID have successfully been matched with the modelling errors present in the model. After manual fixing (left), the WURCS sequence for the glycan failed to return database IDs. Highlighting in red depicts the locations in WURCS notation where both glycans differ.

The new version of Privateer was run on the proposed model. WURCS notations were successfully generated for all glycans, with only 1 glycan chain out of 12 successfully returning a GlyTouCan ID. Under further manual review of the one glycan and with help from other validation tools contained in Privateer, it was found to contain anomer mismatch errors (the three letter code denoting one anomeric form did not match the anomeric form reflected in the atomic coordinates). After the anomer mismatch errors were corrected, the oligosaccharide chain also failed to return GlyTouCan and GlyConnect IDs. The other 11 chains that failed to return a GlyTouCan ID also contained flaws, as described previously (Figure 3).

The analysis of this PDB entry highlights the kind of cross-checks that could be done by Protein Data Bank annotators upon validation and deposition of a new glycoprotein entry. It should be recognised that PDB annotators might not necessarily be experts in structural glycobiology. The fact that these glycans could not be matched to standard database entries should be enough to raise the question with depositors, and at the very least write a caveat on a deposited entry where glycans could not be correctly identified. Furthermore, despite the example showing just *N*-glycosylation, other kinds of glycosylation are searchable as well, and therefore this tool could shed much needed light on the validity of models representing more obscure types of modifications.

#### Example 2 – 2Z62

Successfully matching the WURCS string to a GlyTouCan ID, should not be a sole measure of a structure validity. GlyTouCan is a repository of all potential glycans collected from a set of databases, with the entries often representing glycans. Therefore, the correctness of the composition should be critically validated against the information provided in specialized and high-quality databases such as GlyConnect [56] and UniCarbKB [67]. The computational bridge provides direct search of entries stored in GlyConnect, with plans to expand this to more databases in the near future.

**Figure 4:**
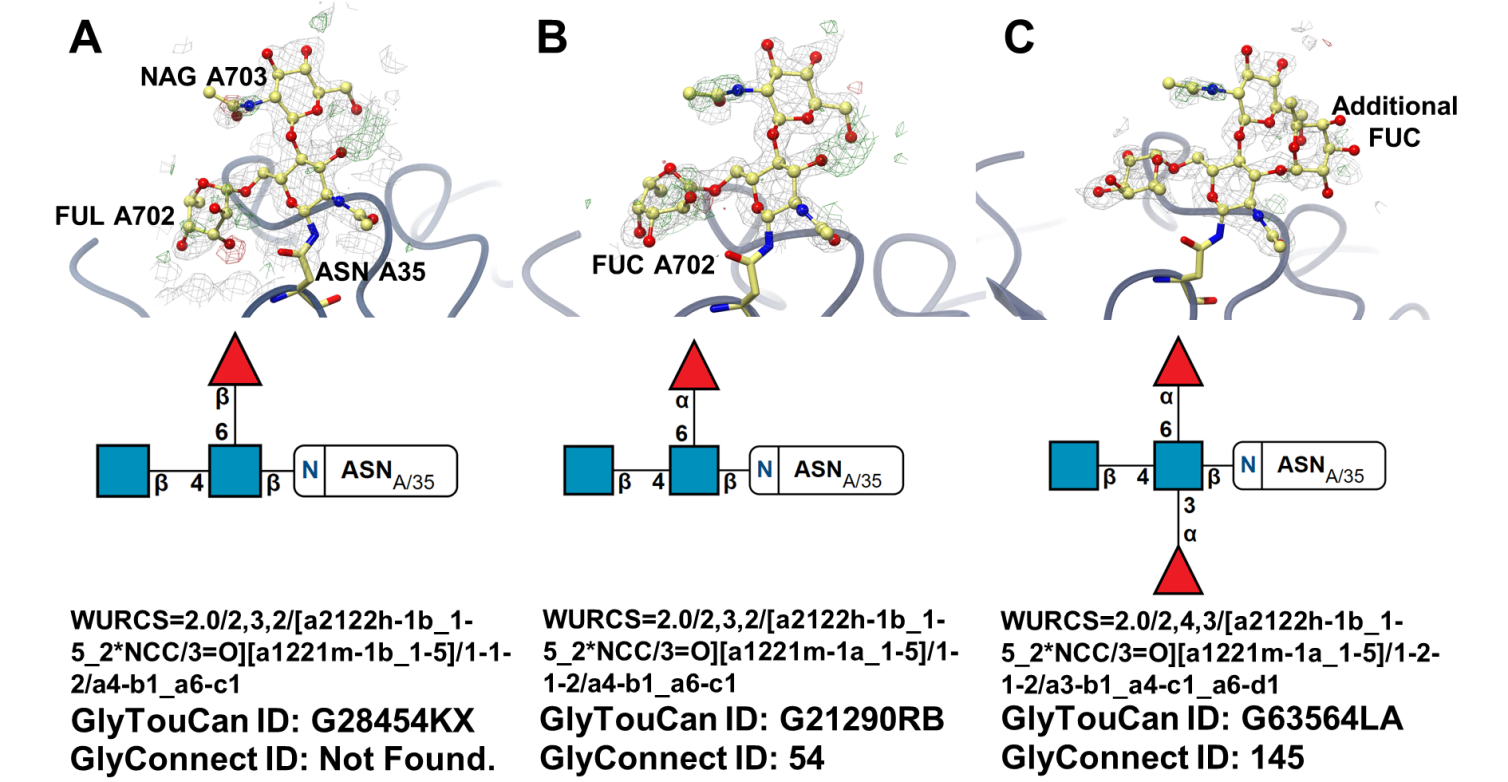
An *N*-linked glycan attached to Asn35 of human Toll-like receptor 4 (A: PDB entry 2z62 [68]). Model iteratively rebuilt by PDB-Redo as shown in steps B and C [41]. Pictures at the top depict glycoprotein models of the region of interest and electron-density maps of the glycan chain (grey: 2mFo DFc map, green and red: mFo DFc difference density map). Pictures at the bottom depict the SNFG representations of glycan chains, their WURCS sequence and accession IDs to relevant databases (taken directly from Privateer's CCP4i2 report).

An example where the sole reliance on the detection of a glycan in GlyTouCan would not be sufficient is rebuilding of the 2Z62 glycoprotein structure [68] to improve the model quality [41] (Figure 4). The analysis of the original model generated the GlyTouCan ID G28454KX, which could not be detected in GlyConnect. The automated tools used by PDB-REDO slightly improved the model by renaming one of the fucose residues from FUL to FUC due to an anomer mismatch between the three letter code and the actual coordinates of the monomer. The new model thus generated the GlyTouCan ID G21290RB, which in turn could be matched to the GlyConnect ID 54. Under further manual review of mFo-DFc difference density map, a (1→3)-linked fucose was added, along with additional corrections to the coordinates of the molecule [41]. The newly generated WURCS notation for the model returned a GlyTouCan ID of G63564LA, with a GlyConnect ID of 145. The iterative steps taken to rebuild the glycoprotein model have been portrayed (Figure 4). Because the data in GlyConnect is approximately 70% manually curated by experts in the field [56], a match of a specific glycan in this database is likely a valid confirmation of a specific oligosaccharide composition and linkage pattern found in nature.

## Conclusion

The mirrors of GlyConnect and GlyTouCan were obtained thanks to the public access to the API commands, which allowed to create scripts that automated the query of the entries stored in the databases with relative ease. However, the integration of additional databases might require support from the developers of those databases. Support for lipopolysaccharides and polysaccharides may be added in future, too, owing to the general purpose of the integrated databases – i.e., they are not limited to protein glycosylation.

Currently, the generated WURCS strings are matched against an identical sequence in the database. This means that if a glycan model has a single modelling mistake, for example, at one end of the chain but is correct elsewhere, the current version of the software would still fail to return a match. This issue has been solved in the development version by the incorporation of a subtree matching algorithm, which will reveal modelling mistakes at specific positions of the glycans, and report these to the user.

Currently, all the developments outlined in this work are accessible exclusively through the Privateer command line interface and through Coot scripts. In order to facilitate the interaction with users, a graphical interface to the new functionality will be provided through the CCP4i2 [38] framework. This new version of the interface is at the testing stage at the time of publication.
